# Evaluation of Sown Cover Crops and Spontaneous Weed Flora as a Potential Reservoir of Black-Foot Pathogens in Organic Viticulture

**DOI:** 10.3390/biology10060498

**Published:** 2021-06-03

**Authors:** Maela León, Mónica Berbegal, Paloma Abad-Campos, Antonio Ramón-Albalat, Tito Caffi, Vittorio Rossi, Gultakin Hasanaliyeva, Pierre Antoine Noceto, Daniel Wipf, Saša Širca, Jaka Razinger, Anne-Laure Fragnière, Patrik Kehrli, Aurora Ranca, Anamaria Petrescu, Josep Armengol

**Affiliations:** 1 Instituto Agroforestal Mediterráneo, Universitat Politècnica de València, Camino de Vera S/N, 46022 Valencia, Spain; maela.leon@uv.es (M.L.); mobermar@etsia.upv.es (M.B.); pabadcam@eaf.upv.es (P.A.-C.); anraal@etsmre.upv.es (A.R.-A.); 2 Dipartimento di Scienze delle Produzioni Vegetali Sostenibili, Università Cattolica del Sacro Cuore, DIPROVES—Crop Protection Area, Via Emilia Parmense 84, 29122 Piacenza, Italy; tito.caffi@unicatt.it (T.C.); vittorio.rossi@unicatt.it (V.R.); gultakin.hasanaliyeva@unicatt.it (G.H.); 3 Agroécologie, AgroSup Dijon, CNRS, INRAE, Pôle IPM-ERL CNRS 6300, BP 86510, 17 rue Sully, CEDEX, 21065 Dijon, France; PierreAntoine.Noceto@inrae.fr (P.A.N.); daniel.wipf@inrae.fr (D.W.); 4 Plant Protection Department, Agricultural Institute of Slovenia, Hacquetova 17, 1000 Ljubljana, Slovenia; sasa.sirca@kis.si (S.Š.); Jaka.Razinger@kis.si (J.R.); 5 Agroscope, Route de Duillier 50, P.O. Box 1012, 1260 Nyon 1, Switzerland; anne-laure.fragniere@agroscope.admin.ch (A.-L.F.); patrik.kehrli@agroscope.admin.ch (P.K.); 6 Calea Bucuresti, No.2, Murfatlar, 905100 Constanta, Romania; director@statiuneamurfatlar.ro (A.R.); cercetare@statiuneamurfatlar.ro (A.P.)

**Keywords:** *Dactylonectria*, *Ilyonectria*, soil-borne fungi

## Abstract

**Simple Summary:**

Black-foot is an important grapevine disease caused by a soil-borne fungal pathogens complex, which are collectively known as *Cylindrocarpon*-like asexual morphs. In organic viticulture, both sown and native cover crop species can act as potential reservoirs of black-foot associated fungi. In our study a wide survey of cover crops grown in organic vineyards was conducted over a diverse range of climatic zones in six different European countries to acquire information about the presence of *Cylindrocarpon*-like asexual morphs on their roots. Several fungal species associated with black-foot disease were found on some of the cover crops evaluated in all the countries. These results provide valuable information for a reasoned choice of cover crop species, or a species mix, that can be used in organic viticulture. This is particularly important for maximizing their benefits and reducing potential problems in vineyards.

**Abstract:**

(1) Background. An extensive survey of grapevine-sown cover crops and spontaneous weed flora was conducted from 2019 to 2020 in organic vineyards in six European countries (France, Italy, Romania, Slovenia, Spain, Switzerland). Our main objective was to detect and identify the presence of *Cylindrocarpon*-like asexual morphs species associated with black-foot disease on their roots. (2) Methods. Fungal isolations from root fragments were performed on culture media. *Cylindrocarpon*-like asexual morph species were identified by analyzing the DNA sequence data of the histone H3 (*his3*) gene region. In all, 685 plants belonging to different botanical families and genera were analyzed. *Cylindrocarpon*-like asexual morphs were recovered from 68 plants (9.9% of the total) and approximately 0.97% of the plated root fragments. (3) Results. Three fungal species (*Dactylonectria* *alcacerensis*, *Dactylonectria torresensis*, *Ilyonectria* *robusta*) were identified. *Dactylonectria* *torresensis* was the most frequent, and was isolated from many cover crop species in all six countries. A principal component analysis with the vineyard variables showed that seasonal temperatures and organic matter soil content correlated positively with *Cylindrocarpon*-like asexual morphs incidence. (4) Conclusions. The presence of *Cylindrocarpon*-like asexual morphs on roots of cover crops suggests that they can potentially act as alternative hosts for long-term survival or to increase inoculum levels in vineyard soils.

## 1. Introduction

Black-foot is an important grapevine disease caused by a complex of soil-borne fungal pathogens belonging to different genera: *Campylocarpon*, *Cylindrocladiella*, *Dactylonectria*, *Ilyonectria*, *Neonectria*, *Pleiocarpon*, and *Thelonectria*, which are collectively known today as *Cylindrocarpon*-like asexual morphs [1,2,3,4]. They are considered the commonest pathogenic fungi associated with young nursery vines/vineyards in many viticultural areas around the world [1,5]. Black-foot significantly impacts grapevine production by compromising the phytosanitary quality of the planting material produced in grapevine nurseries, and the performance of new plantations associated with the young vine decline syndrome [1].

The young vines affected by black-foot disease generally appear normal upon planting, but progressively develop a smaller rootstock diameter, reduced foliage with interveinal chlorosis, and a smaller leaf area over the next 3–5 years. Removing the bark off affected plants reveals black discoloration and necrosis of the basal wood tissues of rootstocks. Below-ground symptoms include low total root biomass, only a few feeder roots, and abundant necrotic root lesions [1,3].

Cover crops can be defined as managed vegetation grown between crop plant rows, including annual and perennial grass species [6]. A recent meta-analysis conducted by Winter et al. [7] concluded that intensive vegetation management in vineyards significantly contributed to providing multiple ecosystem services (ES), such as excellent habitats for pests’ natural enemies, improved carbon sequestration, etc. The planting of cover crops in vineyards might, thus, enhance the soil structure, improve nutrient retention and provision, and increase soil microbial diversity and populations of beneficial microbes [8]. A 3 year study conducted by Diti et al. [9] showed an 85% reduction in soil erosion, a 55% increase in ground water retention, and a 15% improvement in soil carbon sequestration while applying innovative (e.g., cover cropping) soil management in vineyards compared to the traditional system.

Soil-borne fungi and nematodes are relevant damaging agents of grapevines, whose management has been indicated as one of today’s major challenges for a more sustainable viticulture [10,11]. Many publications have reported the beneficial effects of cover crops for controlling nematodes and soil-borne pathogens in vineyards. Diverse cover crop species, either with or without biofumigation properties, have been used to suppress the plant-parasitic nematodes that affect grapevines, such as *Meloidogyne* spp. (root-knot nematode) and *Xiphinema* spp. (dagger nematode) [12,13,14].

Research into soil-borne fungal pathogens has focused on the black-foot disease of grapevines. *Brassica* biofumigation has given promising results in both in vitro and in planta to control black-foot disease pathogens in Australia and New Zealand [15,16]. Berlanas et al. [5] evaluated the effect of white mustard (*Sinapis alba* L.) cover crop residue treatment on controlling black-foot disease in grapevines. These authors found that white mustard biofumigation not only lowered the inoculum of *Dactylonectria torresensis*, but also the incidence and severity of black-foot disease. Vukicevich et al. [17] sampled vineyard sites located in the southern Okanagan Valley (British Columbia, Canada) with different groundcover vegetation and irrigation management systems to investigate effects on *Ilyonectria* spp. abundance. The results showed that *Ilyonectria* spp. increased with the abundance of forbs and exotic species, although only the relation with forbs was consistent across sampling periods. Later, Richards et al. [18] conducted greenhouse experiments to evaluate whether cover crop diversity was able to reduce black-foot disease symptoms and *Ilyonectria liriodendri* abundance in soil by using different combinations of native and common cover crops. When grown alone, white mustard was the only cover crop associated with reduced necrotic root damage in grapevine cuttings cv. Chardonnay and *Ilyonectria* abundance. The suppressive effects of white mustard largely disappeared when paired with other cover crops.

Some of these results indicate that the inoculum of generalist soil-borne plant pathogens, such as *Cylindrocarpon*-like asexual morphs, could build up on certain alternate host plants. Agustí-Brisach et al. [19] isolated black-foot pathogens from the roots of 26 weed species collected in grapevine rootstock mother fields, open-root field nurseries, and commercial vineyards in Spain. Indeed, a reasoned choice of cover crop species or species mix is particularly important in maximizing their benefits and reducing potential problems in vineyards.

Based on these findings, it is necessary to collect further information about the potential of grapevine cover crops, both sown and native species, as alternative hosts for black-foot disease. The present work conducted an extensive survey of grapevine-sown cover crops and spontaneous weed flora from 2019 to 2020 in organic vineyards in six European countries (France, Italy, Romania, Slovenia, Spain, and Switzerland). Our main objective was to detect and identify the presence of *Cylindrocarpon*-like asexual morphs species associated with black-foot disease on their roots. This research work is one of the objectives of the European CORE Organic Cofund BIOVINE project (2018–2021, https://www.biovine.eu (accessed on 1 June 2021). The strategies developed in the BIOVINE project exploit plant diversity in and around vineyards (e.g., cover, hedges) by planting selected plant species to control pests and to promote mycorrhization by, thus, providing organic winegrowers with alternative solutions to pesticides. Our main objective was to acquire new information about the presence of *Cylindrocarpon*-like asexual morphs on the roots of cover crops by accurately identifying them using suitable molecular tools, and to look for potential new fungal species/host combinations and fungal species/country records.

## 2. Materials and Methods

### 2.1. Experimental Plots and Vineyards

Experimental plots were set up in organically managed vineyards in 2019 and 2020 in six European countries (France, Italy, Romania, Slovenia, Spain, and Switzerland) in which diverse sown or native cover crop species were grown according to the project objectives, to investigate pest control improvement, and to evaluate functional biodiversity and the provision of ES. The location and characteristics of these vineyards are shown in Table 1.

### 2.2. Sampling and Fungal Isolation

In each experimental vineyard, the selected cover crop species samples (five plants per sample grown for at least 1.5 months) were collected in summer or autumn (Table 2 and Table 3). For some species, several samples were collected from different subplots in the same experimental vineyards. In 2019, 56 samples were examined (France *n* = 0; Italy *n* = 20; Romania *n* = 12; Slovenia *n* = 4; Spain *n* = 3; Switzerland *n* = 17), as were 81 samples in 2020 (France *n* = 23; Italy *n* = 14; Romania *n* = 8; Slovenia *n* = 4; Spain *n* = 7; Switzerland *n* = 25). This resulted in 137 samples. In the laboratory, the roots of each plant were carefully washed under running tap water to rinse away soil, to then be visually inspected to find evidence of root lesions with necrosis.

In order to isolate *Cylindrocarpon*-like asexual morphs, root fragments were cut only from necrotic areas, which were surface-disinfested for 1 min in 1.5% sodium hypochlorite solution, and washed twice with sterile distilled water. Then, 14 small root pieces were plated per plant on malt extract agar (MEA) supplemented with 0.5 g L^−1^ of streptomycin sulfate (Sigma-Aldrich, St. Louis, MO, USA) (MEAS) (7 fragments for every 2 Petri plates). Plates were incubated for 7–10 days at 25 °C in the dark, and all emerging colonies were transferred to potato dextrose agar (PDA) (Biokar-Diagnostics, Zac de Ther, France).

The preliminary morphological identification of the *Cylindrocarpon*-like asexual morphs colonies was conducted by observing the cultural and microscope characters (mycelium aspect, colony color, conidia type) of the isolates grown on PDA and synthetic nutrient-poor agar (SNA), with or without the addition of two 1 cm^2^ pieces of sterile filter paper on the medium. Petri plates were incubated at 25 °C for 3 weeks under mixed white and near-UV light and with a 12 h photoperiod [1,4].

Then, 93 isolates of the *Cylindrocarpon*-like asexual morphs were selected for the molecular analyses and characterization (Table 2 and Table 3). For this purpose, these isolates were firstly single-spored by the serial dilution method [20]. For long-term storage, the agar plugs with mycelium and the conidia from these cultures were stored in 15% glycerol solution at −80 °C in 1.5 mL cryovials at the fungal collection of the Instituto Agroforestal Mediterráneo of the Universitat Politècnica de València (Spain).

### 2.3. DNA Isolation, Sequencing and Phylogenetic Analyses

For DNA extraction, the fungal mycelium and conidia from the pure cultures grown on PDA for 2–3 weeks at 25 °C in the dark were scraped and transferred to a 2 mL screw-capped conical tube (Thermo Scientific, San Diego, CA, USA) containing four metal 2.38 mm beads (Qiagen, Hilden, Germany) and two tungsten carbide 3 mm beads (Qiagen Hilden, Germany). Total genomic DNA was extracted with the E.Z.N.A. Plant Miniprep Kit (Omega Bio-tek, Doraville, GA, USA) following the manufacturer’s instructions. The homogenization step was performed twice at 5 m/s for 20 s using FastPrep-245G (MP Biomedicals, Santa Ana, CA, USA). DNA was visualized by electrophoresis on 1% agarose gels stained with REALSAFE (REALSAFE Nucleic Acid Staining Solution 20,000×, Durviz S. L., Valencia, Spain) and stored at −20 °C.

In order to identify the *Cylindrocarpon*-like asexual morphs species, partial sequences of the histone H3 (his3) gene region, which is a very informative locus [21], was amplified. PCR amplifications were carried out using 1× PCR buffer, 2.5 mM of MgCl2, 0.2 mM of each dNTP, 0.4 mM of each primer, 1 U of Taq polymerase (Canvax Biotech, S.L., Córdoba, Spain), and 1 μL of template DNA (20 ng/μL). The PCR reaction mix was adjusted to a final volume of 25 μL with ultrapure sterile water (Chromasolv Plus^®^, Sigma-Aldrich, Steinheim, Germany). The Peltier Thermal Cycler-200 (MJ Research) cycle conditions were: 94 °C for 3 min, followed by 35 cycles of denaturation at 94 °C for 30 s, annealing at 60 °C for 30 s, elongation at 72 °C for 45 s, and a final extension at 72 °C for 10 min. The primers used for his3 were CYLH3F and CYLH3R [22]. After confirmation by agarose gel electrophoresis, PCR products were sequenced in both directions by the Macrogen Inc., Sequencing Center (The Netherlands, Europe).

### 2.4. Principal Component Analysis

Experimental vineyards and soil characteristics variables (Table 1), including the *Cylindrocarpon*-like asexual morphs incidence, were subjected to a principal component analysis (PCA) to group the different tested fields and to reduce the observed variables to a smaller number of principal components (artificial variables) to account for most of the variance in the observed variables. The PCA analysis was performed with the Statgraphics Centurion XV (Statgraphics Technologies, Inc., The Plains, VA, USA).

Sequences were assembled and edited to resolve ambiguities, and the consensus sequences for all the isolates were compiled in a single file (Fasta format) with the Sequencher software v. 5.3 (Gene Codes Corporation, Ann Arbor, MI, USA), and were compared to those in the NCBI Genbank database using the Basic Local Alignment Search Tool (BLAST) and a phylogenetic analysis. The GenBank his3 sequences from *Dactylonectria* and *Ilyonectria* reference species were selected based on their high similarity to our query sequences with MegaBLAST. They were added to the sequences obtained and aligned using ClustalW [23]. A maximum parsimony analysis was performed by MEGA X [24] with the tree bisection and reconnection (TBR) algorithm, where gaps were processed as missing data.

## 3. Results

### 3.1. Cylindrocarpon-Like Asexual Morphs Detection and Identification

*Cylindrocarpon*-like asexual morphs were obtained from the roots of the cover crop samples in the vineyards of all the surveyed countries. The isolations on culture media yielded 93 isolates: 34 isolates were obtained in 2019, and 59 in 2020 (Table 2 and Table 3). In all, 685 plants were analyzed, and 9590 root fragments were plated on MEAS. *Cylindrocarpon*-like asexual morphs were recovered from 68 plants (9.9% of the total) and from approximately 0.97% of the plated root fragments.

The color of the colonies of *Cylindrocarpon*-like asexual morphs on PDA varied from white to yellow, or from light to dark brown, with a cottony mycelium. Based on the microscopic observations, all the isolates produced macroconidia and microconidia, as described by Cabral et al. [25], and Agustí-Brisach and Armengol [1]. The DNA sequence data using primers CYLH3F and CYLH3R showed high homologies (≥99%) to the reference sequences in the NCBI Genbank database, which confirmed the identification of the 93 isolates as belonging to species *Dactylonectria alcacerensis* (one isolate), *Dactylonectria torresensis* (90 isolates), and *Ilyonectria robusta* (two isolates) (Table 2 and Table 3).

The fungal species identified in this study were found to be associated with diverse cover and typical vineyard weeds, and also with grass-cover crop genera and species, the most frequent being *Plantago lanceolata* (13 infected plants), *Trifolium repens* (11), *Lolium perenne* (seven), *Taraxacum officinale* (seven), and *Trifolium alexandrinum* (seven). In general, the *Cylindrocarpon*-like asexual morphs isolation showed some preference for plants belonging to the family *Fabaceae*, as 24 isolates (=26%) were recovered in this taxon and several species were infected: *Medicago maculata*, *Pisum sativum*, *Trifolium alexandrinum*, *Trifolium repens*, and *Vicia villosa*. However, they were also quite frequent on *Asteraceae*, *Plantaginaceae* and *Poaceae* species.

Regarding the Cylindrocarpon-like asexual morphs, the species Dactylonectria alcacerensis was recovered only from Plantago lanceolata, Ilyonectria robusta from Pisum sativum and Taraxacum officinale, and Dactylonectria torresensis, the most frequent fungal species, from the other infected cover crops.

### 3.2. Principal Component Analysis

**Figure 1:**
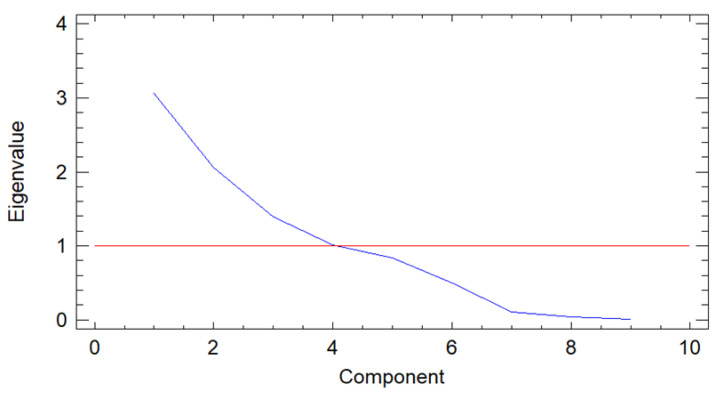
Screen plot of the principal component analysis (PCA) of soil characteristics between eigenvalues and principal components.

The purpose of the PCA was to obtain a few linear combinations of the original variables that account for most data variability. In this case, four components were extracted as four components had eigenvalues over or equaling 1.0. Together they accounted for 83.65% of the variability in the original data (Figure 1).

**Figure 2:**
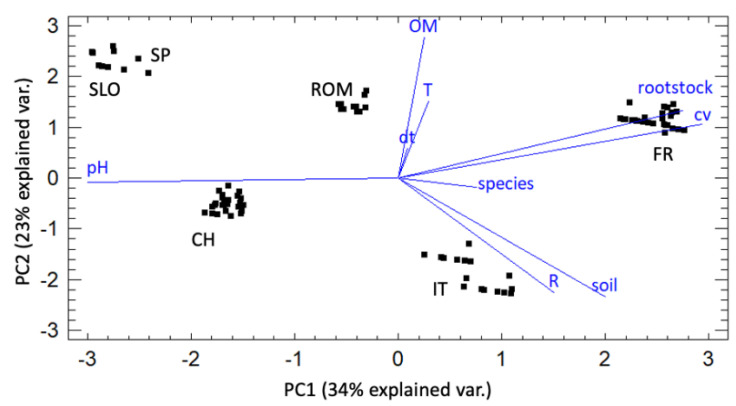
Principal component analysis (PCA) of the six studied vineyards in 2019 and 2020. The percentage values in parentheses correspond to the variance explained by each principal component (dt = *Cylindrocarpon*-like asexual morphs incidence; OM = organic matter soil content; R = total season rainfall; T = seasonal temperatures).

The obtained biplot (Figure 2) shows the data grouped for sampling country, and the effect of the different variables on the first two components selected in the PCA, which together explained 57% of data variability. Seasonal temperatures (T) and organic matter soil content (OM) correlated positively with the *Cylindrocarpon*-like asexual morphs incidence (dt) and strongly affected PC2, while total season rainfall (R) was negatively correlated and affected PC1. The silty-clay loam soils (as characterized in France and Italy) correlated negatively with dt. The rootstocks and cultivar characteristics of the observed vineyards, as well as soil pH did not correlate with the *Cylindrocarpon*-like asexual morphs incidence, despite them strongly impacting PC1.

## 4. Discussion

The present study characterized a large collection of *Cylindrocarpon*–like asexual morphs collected from the roots of cover crop species grown in organic vineyards in different European countries for the first time. Although the percentages of isolation from plants were low, it was not generally difficult to obtain the fungal colonies of the black-foot-associated pathogens from the roots of the diverse cover crop species belonging to the different botanical families and genera included in this study. This confirms current knowledge about these fungal pathogens being ubiquitous because they have been reported in most world grapevine-producing regions, as well as them being saprobes in soil, occurring on dead plant substrates, or acting as latent pathogens or endophytic organisms [1,26]. Black-foot disease caused by *Cylindrocarpon*–like asexual morphs is considered one of the most destructive grapevine diseases in newly established vineyards, mainly due to early infections in grapevine propagation material during the grafting process performed in grapevine nurseries [1,3].

An analysis of DNA sequences allowed three species to be identified, which belong to two genera, namely, *Dactulonectria alcacerensis*, *Dactylonectria torresensis*, and *Ilyonectria robusta*. *Dactylonectria torresensis* was the most frequent species, being isolated from many cover crop species in all six countries. This corroborates previous research findings which have indicated that *Dactylonectria torresensis* is currently considered the most frequent pathogen associated with black-foot disease of grapevine, and has been described in important wine-producing countries, like Australia, Italy, New Zealand, Portugal, Spain, South Africa, and the United States [4,27]. As far as we know, our study is the first report of *Dactylonectria torresensis* in Romania, Slovenia, and Switzerland, and on most of the hosts where this species was found.

Regarding the other less frequent *Cylindrocarpon*-like asexual morph species found in our study, *Dactylonectria alcacerensis* and *Ilyonectria robusta* are also well-known grapevine pathogens associated with black-foot disease of grapevines [28,29]. To the best of our knowledge, our study is the first to report *Dactylonectria alcacerensis* on *Plantago lanceolata*, and of *Ilyonectria robusta* on *Pisum sativum* and *Taraxacum officinale.*

Our study has certain similarities to the previous research carried out by Agustí-Brisach et al. [19], who sampled weeds in grapevine rootstock mother fields, open-root field nurseries, and commercial vineyards in Spain to evaluate them as potential hosts of black-foot pathogens. These authors successfully isolated the species *Cylindrocarpon macrodidymum* from the roots of 15 out of 19 evaluated weed families, and from 26 of 52 weed species. We cannot directly compare our results to those obtained by Agustí-Brisach et al. [19] because the taxonomy of *Cylindrocarpon*-like asexual morphs has been revised several times since its publication. Studies based on multigene phylogeny and morphological comparisons have contributed to describing new genera and species in this group of pathogens [21,25,30], which are currently included in the following genera: *Campylocarpon*, *Cylindrocladiella*, *Dactylonectria*, *Ilyonectria*, *Neonectria*, *Pleiocarpon, Thelonectria* [2,3,4].

A more recent study from Canada has assessed the effect of groundcover vegetation on entomopathogenic fungi (represented by *Beauveria bassiana*) abundance and *Ilyonectria* spp. in vineyards [17]. These authors found that plant community characteristics were related to the fungal abundance for both studied fungi groups. Specifically, *Ilyonectria* spp. increased with the abundance of forbs and exotic species with increasing OM and the use of dual/sprinkler irrigation systems. It is worth pointing out here that, in their study, *Beauveria bassiana* increased with the presence of *Fabaceae* species, similarly to what occurred in our study, with a high isolation rate for the *Cylindrocarpon*-like asexual morphs from the plants belonging to this botanical family, and also with the isolation of *Ilyonectria robusta* from *Pisum sativum*.

The presence of *Cylindrocarpon*-like asexual morphs on roots of cover crops, and spontaneously found weeds and grasses, suggested that they could potentially act as alternative hosts for long-term inoculum survival or to increase inoculum levels in vineyard soils. Work on invasive plant species has evidenced that generalist pathogens, such as *Cylindrocarpon*-like asexual morphs, can build up on exotic species with negligible effects on these plants [31]. Agustí-Brisach et al. [32] detected the presence of *Ilyonectria* spp. and quantified its inoculum on the soil samples collected from commercial nurseries located in the Valencian region (central-eastern Spain) using multiplex nested PCR and quantitative PCR. These authors concluded that the ability to detect and quantify *Ilyonectria* spp. genomic DNA in grapevine nursery soils confirmed that they were important sources of inoculum for black-foot pathogens. More recent research, in which the presence of inoculum of *Cylindrocarpon*-like anamorphs on vineyard soil samples has been evaluated by using semiselective culture media [27] and high-throughput amplicon sequencing and a quantitative PCR approach [33,34], also revealed the abundance of viable propagules of black-foot pathogens in vineyard soils, and the prevalence of *Dactylonectria* and *Ilyonectria* species in the grapevine soil microbiome, respectively.

*Cylindrocarpon*-like asexual morphs readily produce conidia, and some species also produce chlamydospores on culture, which indicates that these propagules are likely to be produced on the diseased roots and stem bases of infected vines. Conidia are dispersed in soil water and chlamydospores can allow these fungi to survive in soil for extended time periods [1,26,27]. Berlanas et al. [27] quantified viable propagules of black-foot disease pathogens in a diverse range of grapevine-cultivated soils and investigated their relation to soil properties. In their study, tested soil physicochemical variables from different fields were subjected to a PCA. The results showed that the inocula of *Cylindrocarpon*-like asexual morphs was present in all soil types, and only a relation was found between calcium carbonate and the colony-forming units of these fungi in soil. Our PCA results fall in line with those obtained by Berlanas et al. [27]. Moreover, an interesting outcome was about the organic matter content in soil, which has not been previously highlighted, and could represent a new factor to be considered when evaluating the risk of black-foot agents in vineyards. This specific aspect should be deeply further investigated to be confirmed. Our results also agree with a previous study that demonstrated that *Cyindrocarpon*-like asexual morphs have abilities to be active in soil over wide pH, temperature, and water potential ranges [35]. These pathogens infect grapevines through natural openings or wounds, such as the non-callused parts of lower trunks. Infection can also occur through wounds in canes, such as disbudding wounds, from which infection progresses downwardly to the base of trunks [26]. In fact, pathogenicity tests conducted by Agustí-Brisach et al. [19] already showed that the black-foot isolates obtained from weeds were able to induce typical black-foot disease symptoms when they were inoculated on grapevine cuttings and could, thus, be a source of inoculum for grapevine infections.

## 5. Conclusions

The results obtained in our study emphasize the importance of selecting the best suited cover crop species for their use in organic viticulture. They can also have implications for previous land use, nursery soil management, or weed management practices in both grapevine nurseries and vineyards. It still remains unclear if the ES provided by cover crop species are not hampered by the promotion of negative plant–soil feedback.

## Figures and Tables

**Table 1:** Characteristics of the experimental vineyards from which the sown or native cover crop species samples were collected.

Country	Location	Cultivar	Rootstock	Age (Years)	Soil Texture	Soil pH	Organic Matter	Rainfall Jan–Oct (mm)		Mean Temperature Jan–Oct (°C)	
								2019	2020	2019	2020
France	Premeaux-Prissey (Burgundy)	Pinot Noir	Teleki 5C	20	silty-clay	5.8	3.0%	--	1060	--	10.1
Italy	Res Uvea farm in Castell’Arquato (Piacenza)	Croatina	Kober 5BB	20	silty-clay-loam	6.9	1.3%	1422	921	10.0	9.8
Romania	Murfatlar vineyard, (Dobrodgea region)	Feteasca neagra	SO4	18	loam	7.9	2.3%	354	400	9.7	9.5
Slovenia	Hruševica, Vinakras (Primorska region)	Refošk	SO4	2	loam	5.3	2.8%	1352	1358	10.0	9.6
Spain	Villar del Arzobispo (Valencia province)	Cabernet Sauvignon	110 Richter	10	clay-loam	8.3	2.6%	223	444	14.1	14.1
Switzerland	Nyon	Chasselas	Rootstock 3309	23	loam	7.8	2.4%	1075	1025	7.5	8.2

**Table 2:** Fifty-six samples of the planted and native cover crop species collected in experimental vineyards in six European countries in 2019, with indications of the *Cylindrocarpon*-like asexual morphs isolated from their roots.

France	Romania (Summer)	Spain (Autumn)	
Not evaluated	*1. Lolium perenne* *2. Lolium perenne* *3. Onobrychis* sp. *4. Onobrychis* sp. *5. Sinapis* sp. *6. Sinapis* sp. *7. Tagetes erecta* L. *8. Tagetes erecta* ***9. Trifolium repens* DT (P1/1; P2/1)** ***10. Trifolium repens* DT (P4/1)** *11. Vicia faba* L. *12. Vicia faba*	*1. Cyperus rotundus* L. *2. Diplotaxis erucoides* (L.) DC. ***3. Salsola kali* L. DT (P1/1)**	
**Italy (Summer)**	**Slovenia (Summer)**	**Switzerland (Summer)**	**Switzerland (Autumn)**
*1. Armoracia rusticana* G. Gaertn., B. Mey. & Scherb. ***2. Armoracia rusticana* DT (P2/1) ^a^** *3. Armoracia rusticana* *4. Lolium perenne* L. *5. Lolium perenne* *6. Lolium perenne* *7. Onobrychis viciifolia* Scop *8. Onobrychis viciifolia* *9. Onobrychis viciifolia* *10. Sinapis* sp. *11. Sinapis* sp. *12*. *Sinapis* sp. *13. Trifolium repens* L. *14. Trifolium repens* ***15. Trifolium repens* DT (P1/1)** *16. Trifolium repens* *17. Trifolium repens* *18. Vicia sativa* L. *19. Vicia sativa* *20. Vicia sativa*	***1. Phacelia* sp. DT (P2/1)** *2. Sinapis alba* L. *3. Trifolium incarnatum* L. *4. Vicia pannonica* Crantz	*1. Bromus tectorum* L. ***2. Geranium columbinum* L. DT (P1/1; P2/1)** *3. Hordeum murinum* L. *4.Lolium perenne* ***5. Plantago lanceolata* L. DT (P1/2; P4/1)** ***6. Trifolium repens* DT (P1/1)**	*1. Bromus tectorum* ***2. Hordeum murinum* DT (P1/2)** ***3. Lolium perenne* DT (P4/1; P5/1)** *4. Lolium perenne* ***5. Medicago maculata* Willd. DT (P3/1; P5/2)** ***6. Plantago lanceolata* DT (P2/1; P4/1; P5/3)** *7. Plantago lanceolata* DT (P1/2) *8. Sanguisorba minor* Scop. ***9. Trifolium repens* DT (P1/5; P2/1)** ***10. Trifolium repens* DT (P3/1)** *11. Veronica persica* Poir.

^a^ The cover crop species in bold indicate a positive isolation of *Cylindrocarpon*-like asexual morphs, which are indicated as follows: DT = *Dactylonectria torresensis* (infected plant number from 5 evaluated plants/number of obtained isolates).

**Table 3:** Eighty-one samples of the planted and native cover crop species collected in experimental vineyards in six European countries in 2020, with indications of the *Cylindrocarpon*-like asexual morphs isolated from their roots.

France (Autumn)	Romania (Summer)	Spain (Summer)	
*1. Arenaria serpyllifolia* L. *2. Avena strigosa* Schreb. ***3. Brassica carinata* L. DT (P4/1) ^a^** *4. Brassica carinata* *5. Geranium* sp. *6. Lathyrus sativus* L. *7. Lens culinaris* Medik. *8. Linum usitatissimum* L. *9.Pisum sativum* L. *10. Pisum sativum* ***11. Pisum sativum* IR (P1/1)** *12. Raphanus sativus* L. *longipinnatus* Bailey ***13. Raphanus sativus longipinnatus* DT (P2/1; P4/1)** *14. Secale cereale* L. ***15. Trifolium alexandrinum* DT (P4/2)** ***16. Trifolium alexandrinum* DT (P1/1; P4/3)** ***17.Trifolium alexandrinum* DT (P1/1; P4/1)** ***18. Trifolium subterraneum* L. DT (P3/1; P5/1)** *19. Vicia faba* *20. Vicia faba* ***21. Vicia villosa* DT (P5/1)** ***22. Vicia. villosa* DT (P2/1; P4/1)** *23. Vicia. villosa*	*1. Lolium perenne* *2. Lolium perenne* *3. Onobrychis* sp. *4. Onobrychis* sp. *5. Sinapis* sp. *6. Sinapis* sp. *7. Tagetes erecta* *8. Tagetes erecta*	*1. Anacyclus clavatus* (Desf.) Pers. *2. Avena sterilis* L. ***3. Cichorium intybus* L. DT (P5/1)** *4. Conyza sumatrensis* (Retz.) E. Walker *5. Plantago albicans* L. ***6. Sonchus oleraceus* L. DT (P3/1)** *7. Xantium orientale* L. *subsp. italicum* (Moretti) Greuter	
**Italy (Summer)**	**Slovenia (Autumn)**	**Switzerland (Summer)**	**Switzerland (Autumn)**
*1. Armoracia rusticana* *2. Armoracia rusticana* *3. Armoracia rusticana* *4. Lolium perenne* *5. Lolium perenne* *6. Onobrychis viciifolia* *7. Sinapis* sp *8. Sinapis* sp ***9. Trifolium repens* DT (P4/1)** *10. Trifolium repens* *11. Trifolium repens* *12. Vicia sativa* *13. Vicia sativa* *14. Vicia sativa*	*1. Raphanus sativus* L. *2. Raphanus* sp. ***3. Rorippa sylvestris* (L.) Bess. DT (P3/7; P5/1)** ***4. Sinapis alba* DT (P5/1)**	*1. Lolium perenne* ***2. Lolium perenne* DT (P3/1; P4/1; P5/1)** *3. Lolium perenne* *4. Lolium perenne* *5. Plantago lanceolata* *6. Plantago lanceolata* *7. Plantago lanceolata* *8. Plantago lanceolata* ***9. Prunella vulgaris* L. DT (P1/1; P4/1; P5/1)** *10. Trifolium repens* ***11. Trifolium repens* DT (P1/1)** *12. Trifolium repens* *13. Trifolium repens*	*1. Lolium perenne* ***2. Lolium perenne* DT (P1/1; P5/1)** *3. Lolium perenne* *4. Lolium perenne* ***5. Plantago lanceolata* DT (P4/1; P5/2)** *6. Plantago lanceolata* ***7. Plantago lanceolata* DT (P1/1; P3/1; P4/2)** ***8. Plantago lanceolata* DA (P4/1) and DT (P1/1)** ***9. Taraxacum officinale* Weber et Wiggers DT (P2/1; P3/1; P4/1; P5/1) and IR (P3/1)** ***10. Taraxacum officinale* DT (P1/2; P2/3; P4/1)** ***11. Trifolium repens* DT (P2/1)** *12. Trifolium repens*

^a^ The cover crop species in bold indicate the positive isolation of *Cylindrocarpon*-like asexual morphs, which are indicated as follows: DA = *Dactylonectria alcacerensis*, DT = *D. torresensis*, IR = *Ilyonectria robusta* (infected plant number from 5 evaluated plants/number of obtained isolates).
